# Identification of the protective mechanisms of Lactoferrin in the irradiated salivary gland

**DOI:** 10.1038/s41598-017-10351-9

**Published:** 2017-08-29

**Authors:** Manabu Sakai, Takumi Matsushita, Ryoko Hoshino, Hitomi Ono, Kazuki Ikai, Takayoshi Sakai

**Affiliations:** 1 Department of Oral-facial Disorders, Osaka University Graduate School of Dentistry, 1-8 Yamadaoka, Suita Osaka, 565-0871 Japan; 2 Department of Clinical Laboratory, Osaka University Dental Hospital, 1-8 Yamadaoka, Suita Osaka, 565-0871 Japan; 3 First Department of Oral and Maxillofacial Surgery, Osaka Dental University, 8-1 Hanazono-cho, Kuzuha, Hirakata Osaka, 573-1121 Japan

## Abstract

Radiotherapy is commonly used in patients with head and neck cancer, and usually results in irreversible salivary glands damage and hypofunction. It is therefore important to manage such irradiation to prevent damage to the salivary glands. A previous study showed that Lactoferrin (LF) has a radioprotective effect, but the mechanism was not determined in salivary glands. In the present study, we investigated the detailed radioprotective effect of LF using both *ex vivo* submandibular salivary gland organ culture and ICR male mice *in vivo*. We found that LF had effects on both cell proliferation and CyclinD1-mediated cell-cycle progression which were regulated via the ERK1/2 and AKT signal transduction pathways. In addition, LF affected acinar cell structure and function after irradiation. These findings suggest that LF may be a useful agent to prevent irradiation effects in salivary glands.

## Introduction

Salivary glands have important functions in maintaining oral health. The major function of salivary glands is to secrete saliva which has many roles, including digestion, lubrication, and supplying minerals, and it has a solvent effect, an antibacterial effect, etc^1^. Head and neck cancer accounts for nearly 5% of all diagnosed malignancies, making it the sixth most commonly diagnosed cancer worldwide. Approximately 500,000 new cases of head and neck cancer are diagnosed each year in the world with the five year survival rates around 50%^2, 3^. Radiation therapy is generally used to treat head and neck cancers. During radiation treatment, the salivary glands are often incidentally irradiated, and irreversible salivary gland damage can occur^4^. While the detailed mechanism of radiation-induced salivary gland damage is unknown, it is hypothesized that irradiation has immediate cytotoxic effects on salivary glands tissue^5, 6^. Hypofunction of salivary glands can cause numerous sequelae, including xerostomia, swallowing problems, chewing and speaking difficulties, taste disorder, altered nutrition, soft-tissue necrosis, microbial distribution change, dental caries, oral mucositis, and esophagitis^4^.

Lactoferrin (LF) is an 80 kDa iron-binding glycoprotein contained in secretions, such as saliva and breast milk. This protein is involved in diverse biological roles, acting as an antimicrobial agent, immunomodulator, an antioxidant, and anti-cancer factor, suggesting that it has a role in host protection^7–9^. Moreover, LF regulates cell growth and differentiation, bone formation, embryonic development, blood leukocyte levels, the proportion of the myelocytic lineage, cell adhesion, and production and release of cytokines^10–14^. The LF receptors are the low-density lipoprotein receptor-related proteins (LRPs) 1, which are multifunctional endocytic receptors involved in the metabolism of various extracellular ligands^15–17^.

Recently, LF has been shown to have radioprotective effects^18^, but the mechanisms for this activity have not been determined. In the present study, we investigated the effects of LF in sublethal irradiation studies on cell proliferation, cell signaling pathways, and alteration of the cell-cycle using both *ex vivo* submandibular salivary gland (SMG) organ culture and salivary glands *in vivo*.

## Results

### LF induces submandibular salivary gland branching morphogenesis in *ex vivo* organ culture

**Figure 1:**
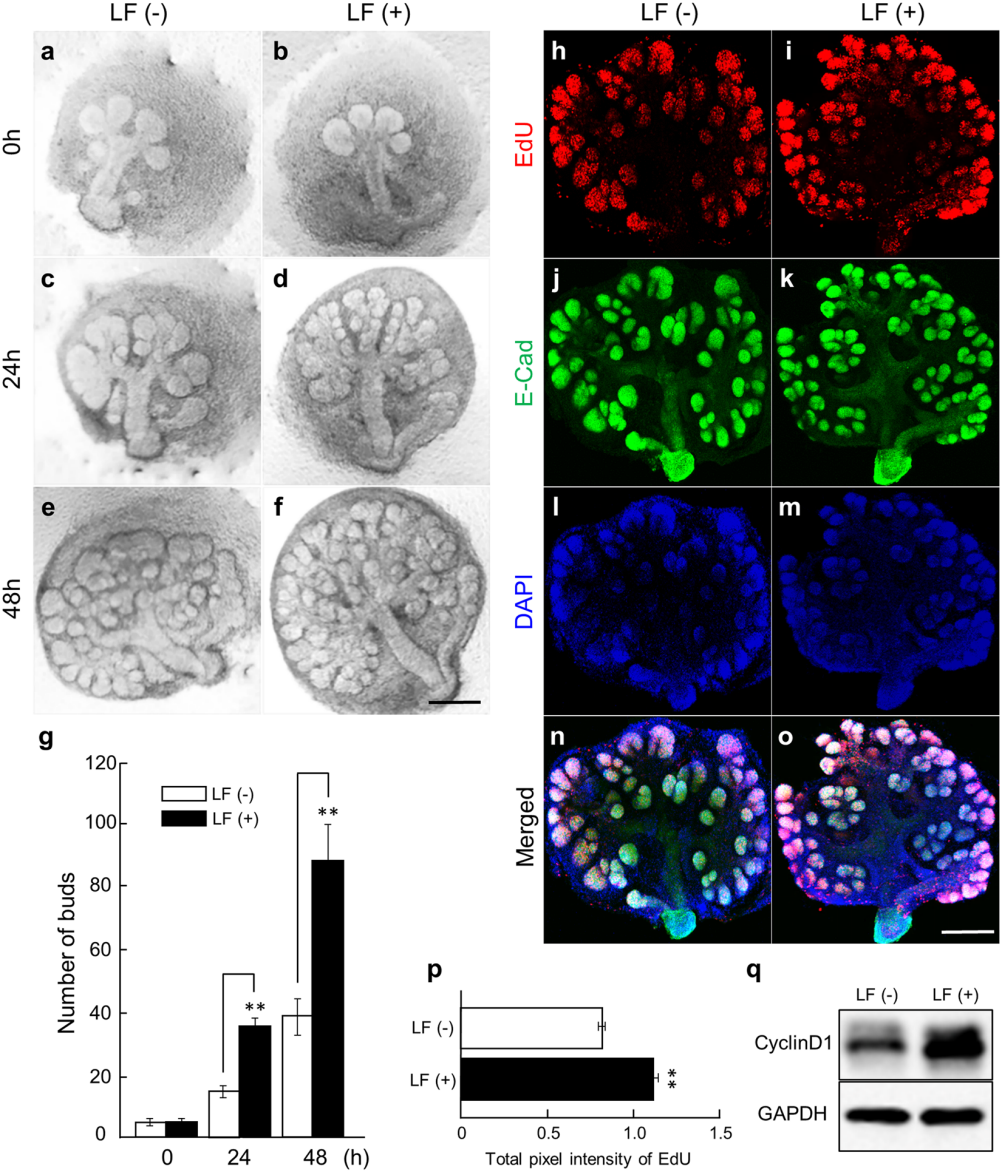
LF enhanced SMG branching morphogenesis in *ex vivo* organ culture. Phase-contrast images show E12.5 + 24 h cultured SMGs at 0, 24, and 48 h of culture with 1.0 mg/ml LF (**a**–**f**). Scale bar: 500 µm. The effects of treatment with 1.0 mg/ml LF were quantified by counting the number of buds per gland at 0, 24, and 48 h (**g**). The 1.0 mg/ml LF-treated groups were positive for EdU as markers of cell proliferation. Proliferating cell nuclei appeared as red punctate spots (**h**,**i**). E-Cadherin was used to stain cell-cell interaction (**j**,**k**). DAPI was used to stain the cell nuclei (**l**,**m**). (**n**): merged picture of (**h**) with (**j**) and (**l**). (**o**): merged picture of (**i**) with (**k**) and (**m**). Scale Bar: 500 µm. The total pixel intensity of EdU at 48 h was quantified using NIH ImageJ software and was normalized to E-Cadherin (**p**). CyclinD1 expression in E12.5 + 24 h cultured SMGs at 48 h of culture with 0.1 mg/ml LF was analyzed by Western immunoblotting (**q**). Bars represent the mean ± SEM. The study was repeated three times, with data shown from a representative experiment. **p < 0.01 compared with control. The gels images were cropped and full-length gels are included in the Supplementary Figure S5.

Since the expression of the LF receptor (LRP1) is high in the early developmental stages (Fig. S1a–c) when the SMG are actively branching, we hypothesized that LF influences embryonic salivary gland development. LF is found in saliva, tears, etc. and especially in high concentrations in milk (mouse: 0.2–2.0 mg/ml and human: 1.0–2.0 mg/ml)^19, 20^. Therefore, we used a range of 0.1–1.0 mg/ml exogenous LF for our embryonic salivary glands studies as pharmacological concentrations to avoid toxic reactions. In addition, other studies have used 0.1–1.0 mg/ml LF *in vitro* ^21, 22^. We added 0.1–1.0 mg/ml of LF to E12.5 (Embryonic day 12.5) SMGs after 24 h of culture (Fig. 1a–f), and the number of buds in the SMG as a measure of branching morphogenesis was counted at 0 h, 24 h, and 48 h after addition of LF to the organ culture medium (Fig. 1g)^23, 24^. These data indicate that the addition of 1.0 mg/ml LF significantly increased the number of epithelial branches in the SMGs. In addition, similar effects were seen with 0.1 mg/ml LF (data not shown).

### LF induces cell proliferation in *ex vivo* organ culture

We found that LF regulates SMG branching morphogenesis, but the mechanism by which this occurs remained unclear. We performed immunohistochemistry for 5-ethynyl-2′-deoxyuridine (EdU) at 48 h after adding LF to the organ culture medium of E12.5 SMGs after 24 h of culture to investigate the effect of LF on SMG cell proliferation (Fig. 1p). LF (1.0 mg/ml) increased the fluorescence intensity of EdU in the SMG (Fig. 1h,i,p). These data indicate that LF induces cell proliferation during SMG branching morphogenesis.

### LF upregulates the expression of CyclinD1 in *ex vivo* organ culture

Previous studies have shown that CyclinD1 has a critical role in cell-cycle regulation during the G1 phase of cell proliferation^25^. The G1 phase is the only stage able to respond to extracellular stimuli, and this important stage determines the cell fate in the cell-cycle. Therefore, we investigated whether CyclinD1 is related to changes in the cell-cycle after LF-treatment. Western blot analysis for CyclinD1 at 48 h after adding LF to the organ culture medium of E12.5 SMGs after 24 h of culture showed that the protein increased (Fig. 1q). These data indicate that LF has effects on cell-cycle progression regulated by CyclinD1 in *ex vivo* SMG organ culture.

### LF increases the expression of phosphorylated ERK1/2 and AKT proteins in *ex vivo* organ culture

**Figure 2:**
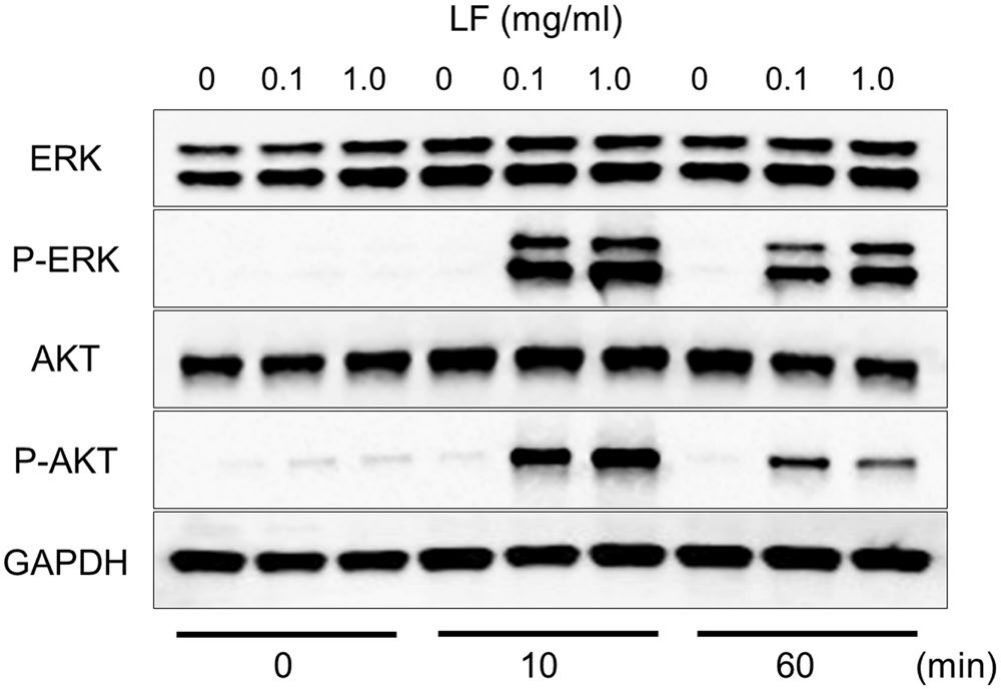
Dose-dependent phosphorylation of LF-induced ERK1/2 and AKT in *ex vivo* organ culture. ERK1/2 and AKT phosphorylation in E12.5 + 72 h cultured SMGs at 0, 10, and 60 min of culture with 0, 0.1, and 1.0 mg/ml LF was analyzed by Western immunoblotting. The study was repeated three times, with data shown from a representative experiment. The gels images were cropped and full-length gels are included in the Supplementary Figure S6.

LRP1 is a cell receptor for diverse proteins, including LF^15–17^. LRP1 is expressed in the SMG at several different developmental stages (Fig. S1a–c). The expression of LRP1 at E13 was significantly higher than that at later stages, such as E15, E17, P1, and P42. Signaling pathways, including the ERK1/2 and AKT pathways, are critically involved in SMG branching morphogenesis^24, 26, 27^. Previous studies have shown that LRP1 was related to ERK1/2 and AKT activation^16, 17, 28^, and that CyclinD1 is regulated by activated ERK1/2 and AKT which then regulates cell proliferation and cell-cycle progression^29–31^. However, the function of LRP1 related to these molecules on salivary glands has not been elucidated. Therefore, we performed western blot analysis for phosphorylated ERK1/2 and AKT after the addition of LF to the organ culture medium of E12.5 SMGs after 72 h of culture. The expression of phosphorylated ERK1/2 and AKT protein increased after 10 min in the presence of LF, then the phosphorylation was decreased at 60 min (Fig. S2). Furthermore, we investigated the LF dose-dependent ERK1/2 and AKT phosphorylation. The SMGs were stimulated for 10 min and 60 min using increasing concentrations of LF (0.1, 1.0 mg/ml), and the expression of phosphorylated ERK1/2 and AKT proteins increased in a dose-dependent manner (Fig. 2). These data indicate that LF has effects on cell proliferation and on cell-cycle progression that are regulated by CyclinD1 via the ERK1/2 and AKT signaling pathways in *ex vivo* SMG organ culture.

### ERK1/2 and PI3K inhibitors affect LF-induced branching morphogenesis in *ex vivo* organ culture

**Figure 3:**
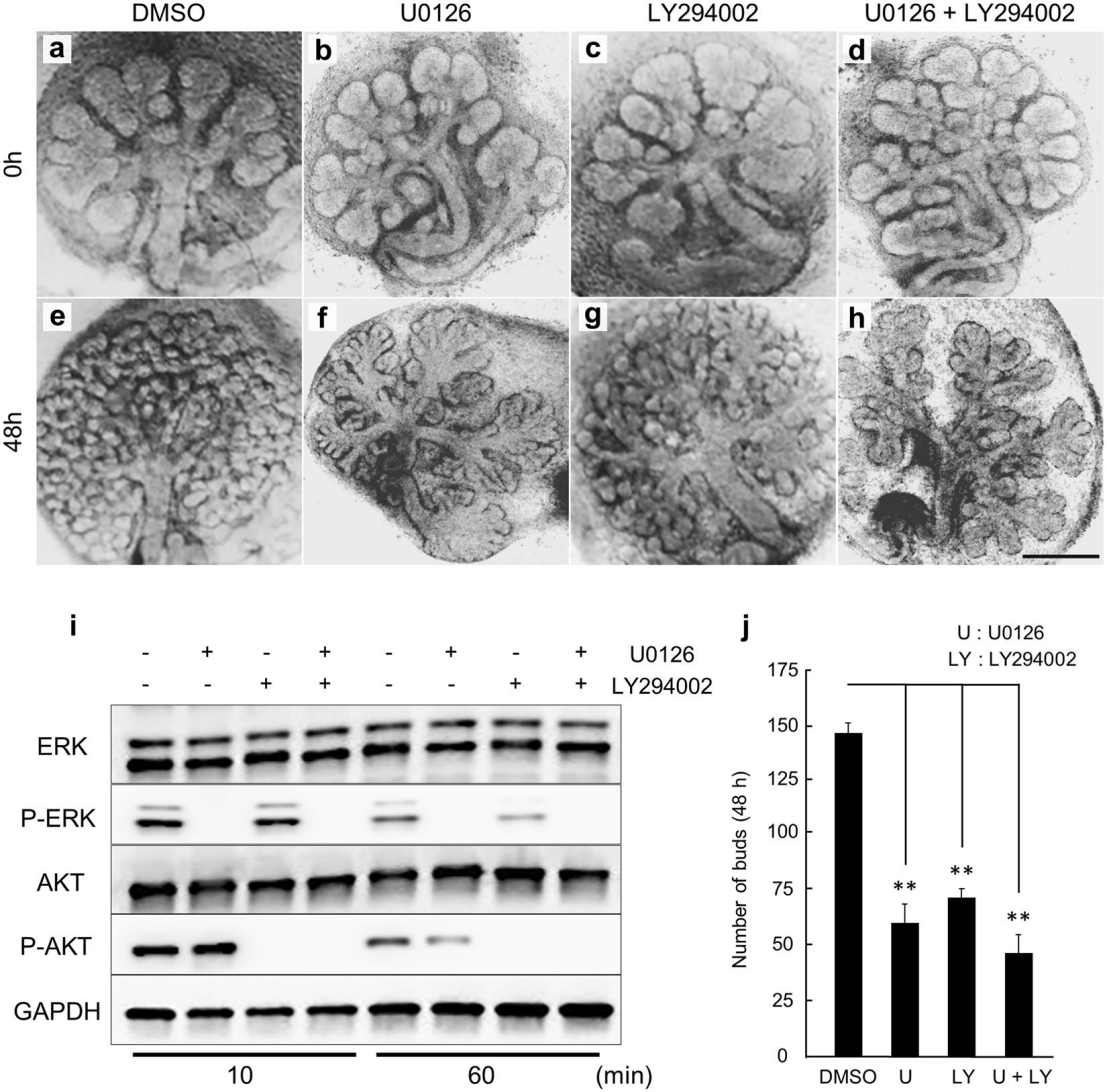
The ERK1/2 inhibitor U0126 and PI3K inhibitor LY294002 inhibited SMG branching morphogenesis and phosphorylation of LF-induced ERK1/2 and AKT in *ex vivo* organ culture. Phase-contrast images show E12.5 + 24 h cultured SMGs at 0 and 48 h of culture with 0.1 mg/ml LF + U0126 and/or LY294002 (**a**–**h**). Scale Bar: 500 µm. ERK1/2 and AKT phosphorylation in E12.5 + 72 h cultured SMGs at 10 and 60 min of culture with 0.1 mg/ml LF + U0126 and/or LY294002 was analyzed by Western immunoblotting (**i**). The effects of inhibitor treatment with 0.1 mg/ml LF were quantified by counting the number of buds per gland at 48 h (**j**). Bars represent the mean ± SEM. The study was repeated three times, with data shown from a representative experiment. **p < 0.01 compared with control. The gels images were cropped and full-length gels are included in the Supplementary Figure S7.

In order to verify whether the ERK1/2 and AKT signaling pathways are required for LF-induced branching morphogenesis or not, an inhibitor experiment was performed. Signal transduction inhibitors (U0126; ERK1/2 inhibitor: 20 µM and/or LY294002; PI3K inhibitor: 20 µM) were added to the culture medium and SMG branching morphogenesis in the presence of the inhibitors in comparison with DMSO-treated control SMGs was evaluated (Fig. 3a–h). In the U0126-treated SMGs, the SMGs showed moderately-branched and elongated epithelial end bud outgrowths. This morphology was remarkably different from the highly-branched epithelial end bud in the DMSO-treated control SMGs (Fig. 3f,j). In the LY294002-treated SMGs, the number of buds was slightly more than those treated with U0126-treated SMGs (Fig. 3j). Compared with the DMSO-treated control SMGs, the bud was similar, and the overall size was very small (Fig. 3g). The combination of both U0126 and LY294002 had a stronger effect on branching morphogenesis (Fig. 3h) and the number of buds was slightly less than each inhibitor alone (Fig. 3j). In addition, western blot analysis of phosphorylated ERK1/2 and AKT after the addition of LF with or without inhibitors was performed on E12.5 SMGs after 72 h of culture. The expression of phosphorylated ERK1/2 and AKT protein decreased after 10 min in the presence of the inhibitors (Fig. 3i). These data indicate that LF affects the process of SMG branching morphogenesis via the ERK1/2 and AKT signaling pathways.

### Irradiation affects submandibular salivary gland morphogenesis in *ex vivo* organ culture

Previous studies have shown that LF has radioprotective effects^18^. The direct additional effects of LF on SMGs were analyzed after irradiation of the organ culture. First, irradiation (2–10 Gy) -treated E12.5 SMGs after 72 h of culture were found to be severely affected by the irradiation in a dose-dependent manner (Fig. S3). In all future studies, 4 Gy irradiation was used, because that dose was most suitable to detect morphological changes in the SMG. After 48 h of culture, the irradiated SMGs had increased intercellular space (Fig. S4a,b). In addition, proliferating cell nuclear antigen (PCNA), which is essential for cellular DNA synthesis and is correlated with the proliferative state of the cell^32, 33^, decreased after irradiation in a dose-dependent manner (Fig. S4c). These data demonstrate that the SMG is sensitive to irradiation, which affected both DNA synthesis and the proliferative state of the cell.

In the irradiated salivary gland, it is likely that all 3 processes of cell death, including apoptosis, necrosis, and autophagy occur^5^. The stage of the embryonic salivary gland development vs adult mouse, irradiation settings, and mouse species, etc. influence the processes of cell death^5, 34–36^. Based on the published studies discussed above, it is difficult to judge accurately the type of cell death produced by irradiation. To clarify the processes of cell death in our experiment, we performed the Terminal deoxynucleotidyl transferase dUTP nick end label (TUNEL) staining to detect DNA fragmentation^37^ and western blot analysis to detect active-caspase-3^38^ after irradiation. After 48 h, irradiated SMGs did not show signs of apoptosis (Fig. S4d, e). Therefore, we believe that our experimental results demonstrate the possibility of necrosis or autophagy.

### Radioprotective effect of LF on submandibular salivary glands in *ex vivo* organ culture

**Figure 4:**
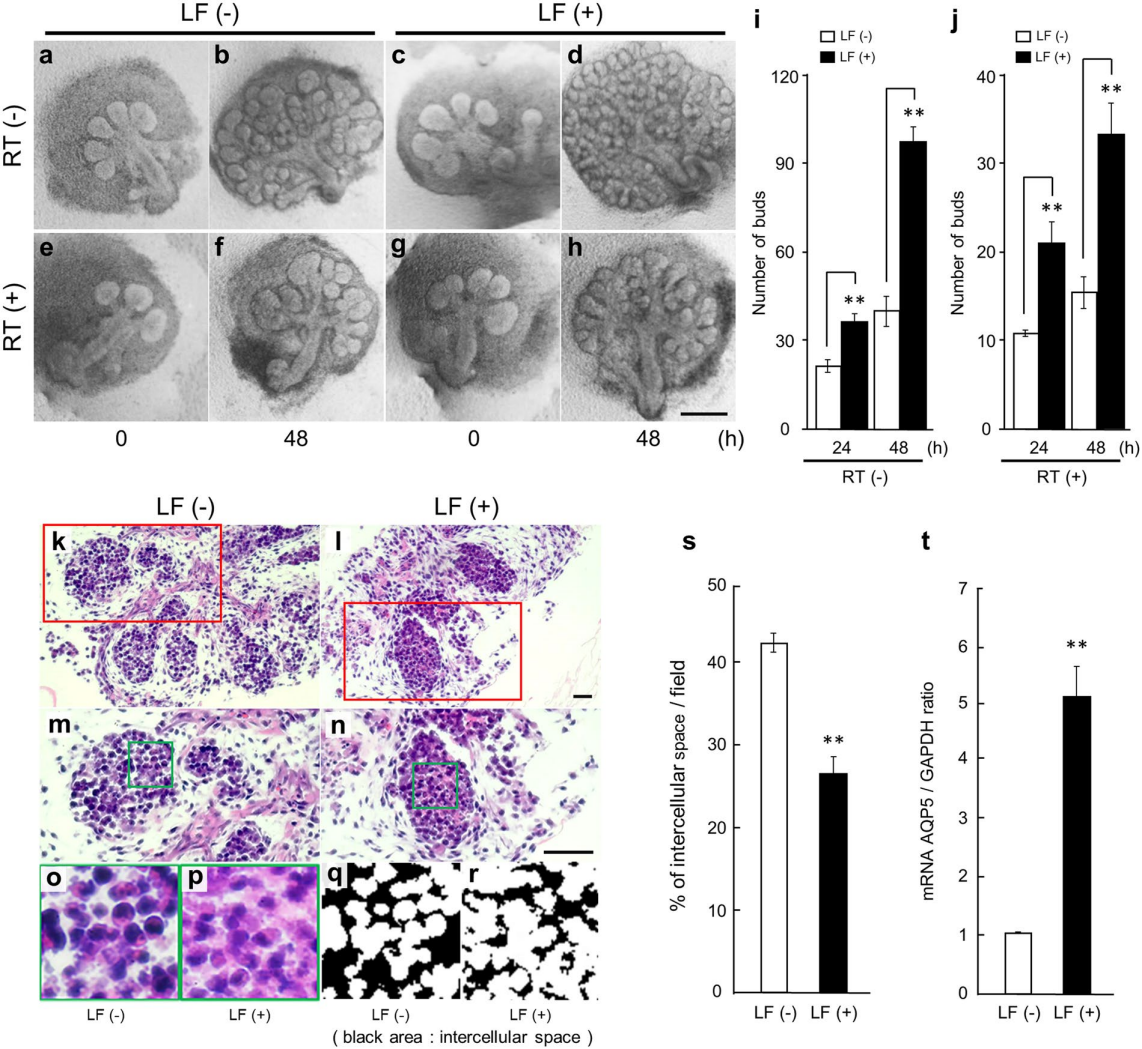
Effect of LF on irradiated SMGs in *ex vivo* organ culture. Phase-contrast images show E12.5 + 24 h cultured SMGs at 0 and 48 h of culture with 0.1 mg/ml LF (**a**–**d**). Phase-contrast images show E12.5 + 24 h cultured SMGs at 0 and 48 h of culture with 0.1 mg/ml LF after irradiation (4 Gy) (**e**–**h**). Scale Bar: 500 µm. The effects of treatment with 0.1 mg/ml LF were quantified by counting the number of buds per gland at 24, 48 h (**i**) and at 24, 48 h after irradiation (**j**). Hematoxylin-Eosin staining images show E12.5 + 24 h cultured SMGs at 48 h of culture with 0.1 mg/ml LF after irradiation (4 Gy) (**k**–**r**). Scale bar: 50 µm. (**m** and **n**) are higher magnifications of (**k** and **l**), respectively. (**o** and **p**) are higher magnifications of (**m** and **n**), respectively. Black area of (**q** and **r**) is intercellular space of (**o** and **p**). Quantification of the percent area per field (50 µm^2^/field) occupied by intercellular space is graphed (s). The expression of AQP5 mRNA in E12.5 + 24 h cultured SMGs at 48 h of culture with 0.1 mg/ml LF after irradiation (4 Gy) was determined by qPCR analysis (t). Bars represent the mean ± SEM. The study was repeated three times, with data shown from a representative experiment. **p < 0.01 compared with control.

Previous studies have shown that LF has radioprotection effects only for lethality^18^, but the detailed mechanism of these effects is unclear. We cultured E12.5 SMGs for 24 h and then added 0.1 mg/ml LF to the culture for 48 h (Fig. 4a–d). In addition, we irradiated (4 Gy) E12.5 cultured SMGs after 24 h of culture and then added 0.1 mg/ml LF to the culture for 48 h (Fig. 4e–h). LF-treated SMGs had an increased number of buds (Fig. 4i), and after irradiation LF-treated SMGs sustained the number of buds (Fig. 4j) in contrast to the LF-non-treated SMGs. Moreover, we researched the possibility that pretreatment of LF before irradiation can increase the effect of LF. We performed the pretreatment with LF at 3 h before irradiation. There was no difference in the results of pretreatment vs after irradiation (data not shown). Pretreatment of LF did not work more effectively for radioprotection. Therefore, these results lead us to believe that LF should be injected immediately before or after irradiation to have its radioprotective effects. Next, the intercellular space and the expression of AQP5 (acinar cell maker) were evaluated in the LF-treated or LF-non-treated SMGs after irradiation. Previous studies showed that AQP5 can be used as a marker of acinar cell structure and function in salivary glands^33, 39, 40^. LF-treated SMGs had a decrease in intercellular space (Fig. 4k–s) and an increase in the expression of AQP5 (Fig. 4t) relative to the LF-non-treated SMGs. These data show that LF maintained acinar cell structure and had radioprotective effects in *ex vivo* SMG organ culture.

### Radioprotective effect of LF on salivary glands *in vivo*

**Figure 5:**
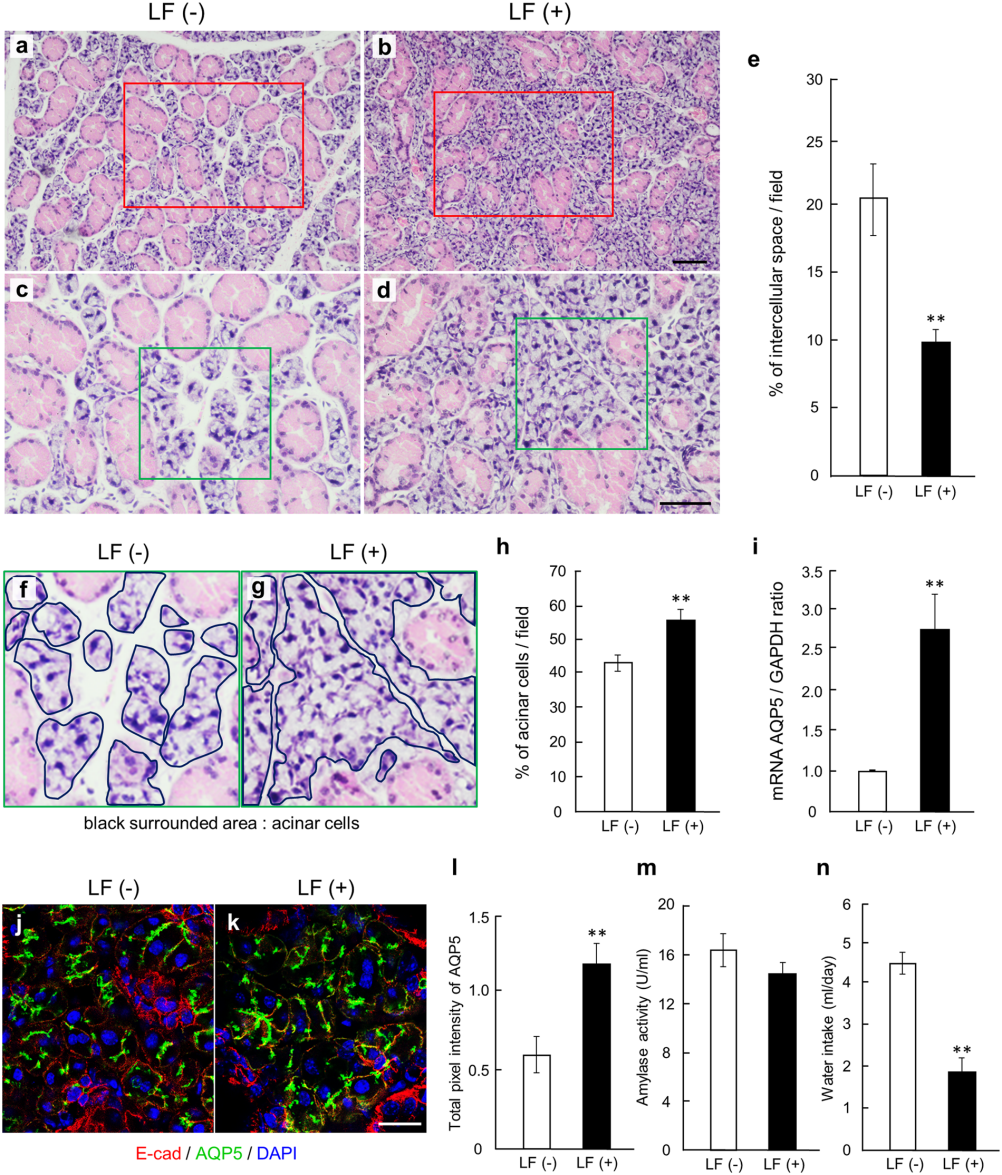
Effect of LF on irradiated salivary gland *in vivo*. Hematoxylin-Eosin staining images show salivary glands from 6-week mice treated with 4.0 mg/animal LF at 1 week after irradiation (9 Gy) (*n* = 6) (**a**–**d**). Scale bar: 100 µm. (**c** and **d**) are higher magnifications of (**a** and **b**), respectively. (**f** and **g**) are higher magnifications of (**c** and **d**), respectively. Quantification of the percent areas per field (250 µm^2^/field) occupied by intercellular space and by acinar cells is graphed (**e** and **h**) (*n* = 3). The expression of AQP5 mRNA in 6-week mice treated with 4.0 mg/animal LF at 1 week after irradiation (9 Gy) was determined by qPCR analysis (*n* = 9) (**i**). Immunofluorescent images show the localization of AQP5, E-Cadherin, and DAPI in salivary glands from 6-week old mice treated with 4.0 mg/animal LF at 1 week after irradiation (9 Gy) (*n* = 4) (**j**,**k**). Scale bar: 50 µm. The total pixel intensity of AQP5 at 48 h was quantified using NIH ImageJ software and was normalized to E-Cadherin (**l**). Salivary amylase activity was examined by the Assay Kit from 6-week mice treated with 4.0 mg/animal LF at 1 week after irradiation (9 Gy) (*n* = 3) (**m**). Water intake was examined using water supply bottles from 6-week mice treated with 4.0 mg/animal LF at 1 day after irradiation (9 Gy) (*n* = 12) (**n**). Bars represent the mean ± SEM. **p < 0.01 compared with control.

A recent study used 4.0 mg/animal LF (the final concentration in blood: 1.7 mg/ml) for radioprotection against lethality^18^. ICR male mice was used to rule out the possibility of native LF effect because LF is major protein induced by estrogen cycle in female mice^41^. Mice (6–8 weeks) were treated with whole-body irradiation at 9 Gy followed by the intraperitoneal administration of either LF (4.0 mg/animal) or saline as control. At 1 week after irradiation, the mice were sacrificed, and the organs were collected. The 1 week time interval was chosen since previous studies found that the mice die after 10 days^18^. Striking morphological changes were observed in the SMGs (Fig. 5a,b). We mainly found the loss of acinar area instead of interstitial fibrous connective tissue and lymphocytic infiltration in the irradiated SMG. Previous studies showed that acute physiological response after irradiation is loss of acinar cell, flow rate, etc^5^. We studied acute response to radiation, rather than the more-common long-term hyposalivation seen in other studies. As seen in the *ex vivo* organ culture, the SMGs from the LF-treated mice had decreased intercellular space (Fig. 5e) and the size of the acinar cells was maintained (Fig. 5h). Previous studies using DAB-colored staining on sliced sections found that irradiation altered AQP5 localization and reduced AQP5 expression in the irradiated SMGs^42^. However, in fluorescent confocal images, AQP5 localization was found to be similar in both LF-treated and LF-non-treated mice (Fig. 5j,k). Next, we measured both mRNA and fluorescence intensity to detect the AQP5 expression level. We prepared immunofluorescence images to investigate the fluorescence intensity of AQP5 as previous described^43, 44^. Finally, we found that AQP5 mRNA expression (Fig. 5i) and fluorescence intensity (Fig. 5l) of the LF-treated SMGs were higher than that in the LF-non-treated SMGs. This result indicates that LF affects only the expression of AQP5 and not the localization.

We then evaluated the function of the LF-treated SMGs after irradiation. At first, we examined amylase activity after irradiation. Since a previous study showed that amylase activity rises after irradiation^45^, we checked the amylase activities of the nonirradiated control mice in addition to the irradiated mice. There was no significant difference in amylase activity between nonirradiated control mice and irradiated mice (data not shown). Next, we investigated the amylase activities of LF-non-treated mice and LF-treated mice after irradiation. There was not a significant difference in amylase activities (Fig. 5m). The serous acini are considered to be the most radiosensitive, followed by the mucous acini. The acute effects of irradiation after 1 week did not completely destroy the serous acini^35^. In addition, daily fluctuations are observed in the amylase concentration after irradiation^45^, so it may be difficult to accurately measure amylase activity.

Second, we examined the amount of drinking water after irradiation. Reduced saliva flow and reduction in water secretion are observed as acute side effects of irradiation^34, 45^. Irradiated LF-non-treated mice drank more water than the LF-treated irradiated mice (Fig. 5n). Acute irradiation did not change amylase activities (Fig. 5m). However, acute effects can include oral mucositis and dry mouth^46^. Although we actually did not directly observe oral mucositis in this experiment, it seems that there are some stomatitis symptoms due to irradiation described in many reports^5, 46, 47^. These symptoms could make the LF-non-treated mice increase their water consumption more than the LF-treated mice. This result indicates that the LF-non-treated mice had more irradiation damage and increased their water intake because LF is radioprotective. These data indicate that LF has radioprotective effects on salivary glands *in vivo* (Fig. 5a–l,n).

## Discussion

There are many intracellular signaling cascades activated by cytokine and growth factor receptors, and their ligands are known to participate in SMG branching morphogenesis^26, 27^. In the present study, we examined the role of signaling cascades induced by LF^19, 20^, which is known to trigger intracellular signaling via LRP1^15, 16^. Involvement of LF-related signaling has not been documented for the process of branching morphogenesis in the fetal SMG. In this study, we found that LF induces SMG branching morphogenesis accompanied by cell proliferation using an *ex vivo* organ culture. Furthermore, ERK1/2 and AKT signaling pathways are involved in this LF-induced SMG branching morphogenesis. The chemical inhibitor studies revealed that ERK1/2 signaling is mainly related to the formation of end buds, and AKT signaling effects the process of cell growth. Both these signaling pathways triggered by LF are important for SMG branching morphogenesis. Though SMG branching morphogenesis after the single-dose of LF had lasted at least 48 h, we couldn’t detect ERK1/2 and AKT phosphorylation at 24 h and 48 h. In this study, the single-dose of LF was enough to affect SMG branching morphogenesis without the need for multiple-dose of LF and continuous phosphorylation of the ERK1/2 and AKT. If we evaluated multiple-dose of LF, we might be able to detect these phosphorylation at 24 h and 48 h.

CyclinD1, a component of the core cell-cycle system, is a regulator of cell-cycle progression and can function as a transcriptional co-regulator. We showed that CyclinD1 expression increases at 48 h after the adding LF. Since CyclinD1 expression depends on ERK1/2 and AKT signaling pathways^29–31^, CyclinD1-mediated cell-cycle progression may be regulated by these signaling pathways in SMG branching morphogenesis. ERK1/2 and AKT activation increased at only 10 min after adding LF, but the increased CyclinD1 expression was later than that time. In fact, cell-cycle progression for SMG branching morphogenesis is thought to begin soon after adding LF, because LF has already started to promote SMG branching morphogenesis at 24 h. But, we couldn’t know the detailed reason why there is a long-time lag to detect the increased CyclinD1 expression. This our research is the first attempt using an *ex vivo* SMG organ culture to find out the relationships between the CyclinD1 and the ERK1/2 and AKT signaling pathways. Though *ex vivo* SMG organ culture is more utility system than cell line culture *in vitro* to detect normal biological reaction, this culture system is very complex. Accordingly, a long-time lag may occur due to *ex vivo* SMG organ culture with a complicated mechanism. Further studies are needed to define the most suitable dose of LF and clarify the detailed mechanism of signaling pathways underlying LF radioprotection in the salivary gland.

Recent research indicated that LF has a radioprotective function after irradiation exposure^18^, but its mechanisms have not been clarified. In this study, we first showed that LF has a radioprotective function both *ex vivo* SMG organ culture and salivary glands *in vivo*. Though acinar cell damage and morphological change of end buds were observed after irradiation, LF maintained tissue stabilization/protection. Previous studies showed that ERK1/2 and AKT signaling pathways are involved in radioprotection^48, 49^. Since we showed that LF induces activation of these signaling pathways, radioprotective ability of LF may be due to this mechanism. In addition, we suspect another mechanism of radioprotection. Irradiation exposure results in a cell-cycle delay^50^. The G1 phase is the only stage able to accept extracellular stimuli and this important stage determines the fate of a cell in the cell-cycle. CyclinD1 plays a significant role in controlling cell-cycle progression through the G1 phase^25^. In this study, we showed that LF-induced CyclinD1 up-regulation might be regulated by ERK1/2 and AKT signaling pathways^29–31^. Since the growth factor restarts proliferation to cell-cycle delayed cells by promoting the transition from Go/G1 to S-phase^25^, LF may induce the radioprotective function to progress CycinD1-mediated cell-cycle via these signaling pathways.

It has been reported that LF has a hydroxyl radical-scavenging activity^18, 51^. Irradiation splits water molecules in living cells, generating reactive free radicals (hydroxyl radicals: ·OH etc.) which cause severe damage to DNA, proteins, and amino acids that are essential components for living organisms^52^. If oxidative stress is weak, the cell can survive by its own repair function, but in case of more serious stress, the cell goes to death. Previous studies showed that LF inhibits the fenton reaction through chelating iron to prevent oxidative reaction^18^ or works as a sacrificial scavenger for reactive oxygen species^51^. These hydroxyl radical-scavenging activities would interact with the radioprotective function of LF which we clarified this research. Previous studies showed that UV irradiation degrades LF molecule^51^. Though the irradiation system we used might decrease native LF, we added a high concentration LF immediately after irradiation. As a result, the SMG may prevent damage from irradiation and/or be able to recover instantaneously due to the effects of LF.

Finally, we propose the possibility of two functions of LF toward the future: First, LF may be a radioprotective agent to block irradiation damage. Second, LF may be a radiorecovery promoting agent after irradiation damage. Further studies are needed to clarify the detailed mechanism of anti-irradiation function related to LF. This function could eventually lead to the clinical applications for future.

## Methods

### Submandibular salivary gland culture

All animal experiments were carried out in strict accordance with the recommendations in the Guide for the Care and Use Committee of the Osaka University Graduate School of Dentistry, Osaka, Japan. The protocol was approved by the Committee on the Ethics of Animal Experiments of Osaka University Graduate School of Dentistry (permit number: 25-004-0). All surgeries were performed under sodium pentobarbital anesthesia, and all efforts were made to minimize animal suffering. The SMGs were isolated from embryonic ICR mice (Japan SLC, Inc. Hamamatsu, Japan) and cultured on Nucleopore membranes (1 µm pore size, GE Healthcare UK Ltd, Buckinghamshire, England) in serum-free Dulbecco’s modified Eagle’s medium/F12 medium, as previously described^53^. After 1 day, the media were replaced with fresh media containing 0.1–1.0 mg/ml bovine Lactoferrin (LF) (Morinaga Milk Industry, Co. Ltd, Tokyo, Japan). LF was dissolved in serum-free medium to the desired concentrations.

### Cell proliferation assay

E12.5 SMGs were cultured in medium either with or without LF for 48 h. Cell proliferation was measured using a Click-iT Plus EdU Alexa 647 Fluor Imaging Kit (Life Technologies, Gaithersburg, MD, USA) following the commercial protocol. The SMGs were incubated with 10 μM EdU at 37 °C for 3 h. They were fixed in 3.7% formaldehyde for 15 min, washed twice with 3% bovine serum albumin in PBS, incubated with 0.5% Triton X-100 in PBS for 20 min, and then the Click-iT reaction cocktail was added at 37 °C for 30 min. After washing in PBS, the SMGs were incubated with Blocking buffer (PBS containing M.O.M. (Mouse Ig Blocking Reagent, Vector Laboratories, Inc., Burlingame, CA, USA) and 5% donkey serum) and then with primary antibody in diluent (PBS containing 8% protein concentrate; M.O.M. Kit, Vector Laboratories) overnight at room temperature. Specific antibody were used: anti-E-Cadherin (1:200, R&D Systems Inc., Minneapolis, Minnesota, USA). After washing with PBS containing 0.5% Tween 20 (PBST), the tissues were incubated with Cy2-labelled donkey anti-goat IgG (1:100, Jackson Immuno Research Laboratories Inc., West Grove, Pennsylvania, USA) for 2 h at room temperature in diluent (PBS containing 8% protein concentrate). The SMGs were then washed in PBS and incubated with DAPI (1:500, Thermo Scientific, Waltham, MA, USA). The coverslips were mounted on glass slides by inverting them into mounting solution, ProLong Gold antifade reagent (Life Technologies). Immunofluorescence was examined using SP8 confocal microscopy (Leica, Wetzlar, Germany). EdU incorporation at 48 hours was detected with fluorescently labeled EdU. The proliferating cell nuclei appear as red punctate spots. The images are compressed stacks of optical sections through the entire gland. The fluorescent EdU staining of the whole gland was quantitated using the ImageJ analysis program (National Institutes of Health, Bethesda, MA, USA), as previously described^43, 44^.

### Western Blot Analysis

The SMGs at E 12.5 and E13 were lysed with RIPA buffer (nacalai tesque, Kyoto, Japan) supplemented with protease and phosphatase inhibitors (nacalai tesque). Cell lysates were centrifuged (15,000 × g) for 10 min, and the supernates were heated at 95 °C for 5 min in denaturing Laemmli buffer (Bio-rad Laboratories, Inc. Hercules, CA, USA). Proteins were separated by SDS-PAGE and transferred to Polyvinylidene difluoride (PVDF) membranes (Bio-rad Laboratories). The membranes were blocked with 3% low fat milk in Tris-buffered saline containing Tween 20, and then incubated with either anti-AKT (1:1,000, Cell Signaling Technology, Beverly, MA, USA), anti-Phospho-AKT (1:1,000, Cell Signaling Technology), anti-ERK1/2 (1:1,000, Cell Signaling Technology), anti-Phospho-ERK1/2 (1:1,000, Cell Signaling Technology), anti-PCNA (1:5,000, BD, San Diego, CA, USA), anti-CyclinD1 (1:10,000, abcam, Cambridge, MA, USA), anti-LRP-1 (1:20,000, abcam), and anti-GAPDH (1:1,000, Cell Signaling Technology). The bound antibodies were detected with anti-rabbit IgG, HRP-linked antibody (1:1,000, Cell Signaling Technology), anti-mouse IgG, HRP-linked antibody (1:1,000, Cell Signaling Technology), and the ECL detection kit (Bio-rad Laboratories).

### Inhibitor treatment

Signal transduction inhibitors (Cell Signaling Technology) were resuspended in DMSO, and added to serum-free medium at the indicated concentrations. At least six SMGs were used for each treatment for each experiment. Inhibitors were assayed at concentrations, as previously described^27, 54, 55^. Control medium contained vehicle at the same concentration as was included with the inhibitor. The SMGs were pretreated with U0126 and/or LY294002 for 1 h, and then LF was added to medium. For immunoblotting, the SMGs were collected at 10 min and 60 min after LF addition. Photographs were taken at 0 h and 48 h with a digital SLR camera (Fuji FinePix, Fuji, Tokyo, Japan) fitted on an Axiovert 25 microscope (Carl Zeiss).

### Quantitative real-time reverse transcription-polymerase chain reaction

The SMGs and 6-week mouse salivary glands were evaluated. Total RNA was isolated from embryonic tissues using TRIzol Reagent (Life Technologies) according to the manufacturer’s instructions, and was treated with PureLink® DNase Set (Thermo Scientific) to avoid genomic DNA contamination. For cDNA synthesis, reverse transcription was performed using the Prime- Script RT Reagent Kit (Takara Bio Inc., Otsu, Shiga, Japan). Quantification of PCR products was performed using the MyiQ Single-Color Real-Time PCR Detection System (Bio-Rad Laboratories) with iQ SYBR Green Supermix (Bio-Rad Laboratories). The amplification program comprised 40 cycles of denaturation at 95 °C for 5 s, annealing at 55 °C for 20 s, and extension at 72 °C for 20 s. The qPCR results for each sample were normalized with glyceraldehyde-3-phosphate dehydrogenase (Gapdh). The results were expressed as normalized ratios. The primer sequences used were as follows:

AQP5: forward 5′-GAGGACTGGGAAGATCATAGAGAGG-3′ and reverse 5′-CAAACTCTTCGTCTTCCTTTTCTCC-3′

Gapdh: forward 5′-CCATCACCATCTTCCAGGAG-3′ and reverse 5′-GCATGGACTGTGGTCATGAG-3′

LRP-1: forward 5′-GTGCTTCAATGGTGGTAGTTGTTTC-3′ and reverse 5′-AGCTCACACTTATCGCCTGTGTAAC-3′.

### Immunofluorescence microscopy

Paraffin-embedded SMGs and 6-week mouse salivary glands were evaluated. Tissue sections were de-paraffinized, and antigen retrieval was performed by autoclave heating (instant antigen retrieval H buffer, 121 °C for 5 min). After washing in PBS, the samples were first incubated with Blocking buffer (PBS containing M.O.M. (Mouse Ig Blocking Reagent, Vector Laboratories) and 5% donkey serum) and then with primary antibody in diluent (PBS containing 8% protein concentrate; M.O.M. Kit; Vector Laboratories) overnight at room temperature. Specific antibodies were used: anti-AQP5 (1:200, Alomone Laboratories, Jerusalem, Israel), anti-E-Cadherin (1:200, R&D Systems), and anti-LRP-1 (1:100, abcam). After washing with PBS containing 0.5% Tween 20 (PBST), the tissues were incubated with Cy2-labelled donkey anti-goat IgG (1:100, Jackson Immuno Research Laboratories) and with Alexa647-labelled donkey anti-rabbit IgG (1:100, Jackson Immuno Research Laboratories) for 2 h at room temperature in diluent (PBS containing 8% protein concentrate). The tissues were then washed in PBS and incubated with DAPI (1:500, Thermo Scientific). The coverslips were mounted on glass slides by inverting them into mounting solution, ProLong Gold antifade reagent (Life Technologies), and examined by SP8 confocal microscopy. Fluorescence Images were processed with ImageJ software.

### Histological Analysis

After deparaffinization and rehydration of paraffin-embedded SMGs and 6-week mouse salivary glands, tissue sections were cut at 4 μm thickness and stained with hematoxylin and eosin. The area of intercellular space and acinar cells were measured by ImageJ software. At least three fields of buds were evaluated.

### Mice Treatment

ICR male mice aged 6 weeks (Japan SLC) were maintained under conventional clean conditions and provided with commercial pellets, MF (Oriental Yeast Co. Ltd, Tokyo, Japan), and water. Bovine LF was dissolved in 0.3 ml of saline to prepare the concentration of 4.0 mg/animal. To induce IR-induced dysfunction, animals were restrained and treated with whole-body X-ray irradiation at 9 Gy using an MX-80 Labo X-ray (MediXtech, Japan). Saline containing LF was intraperitoneally injected into mice immediately after irradiation. As a control group, saline was intraperitoneally injected into the remaining mice. After irradiation, both groups were maintained with commercial pellets and after 1 week the mice were sacrificed.

### Amylase activity assay

Mice were anesthetized using sodium pentobarbital (50 mg/kg). Saliva production was stimulated by intraperitoneal injection of pilocarpine (0.1 mg/kg). Salivation generally occurred in less than 20 s. Saliva was collected for 30 min in 1 min intervals using a suction apparatus (Ringcaps, Hirschmann Laborgera GmbH & Co.KG, Eberstadt, Germany). Mice were positioned on their side with head slightly down to facilitate suction. The amylase activity of secreted saliva was measured using the α-Amylase Assay Kit (Kikkoman Corp., Chiba, Japan) with 2-chloro-4-nitrophenyl 6^5^-azido-6^5^-deoxy-β-maltopentaoside (N3-G5-β-CNP) as the substrate. The production of 2-chloro-4-nitrophenol (CNP) was measured using a Nano Drop 2000C (ThermoScientific). One unit of α-amylase activity was determined as the release of 1 μmol of 2-chloro-4-nitrophenol within 1 min.

### Measurement of water intake

After 9 Gy irradiation, LF-injected mice and saline-injected mice were housed separately in plastic cages with free access to food and water. Water consumption were measured using a water supply bottle (Shinano manufacturing Co.,Ltd. Tokyo, Japan) for daily drinking water measurement for 5 days. The average volume of water per mouse per day was calculated.

### Statistical Analysis

The Student’s *t*-test was used to determine the *P*-value for statistical significance. Bonferroni’s multiple comparison post-test was combined with ANOVA (One-way analysis of variance) to compare differences between the means within an experiment. *P* < 0.05 was considered significant. Results are expressed as the means ± S.E.

## Electronic supplementary material

Supplemental Information
