# Supplementary material for: Identification of the protective mechanisms of Lactoferrin in the irradiated salivary gland
Source: Sci Rep. 2017 Aug 29;7:9753. doi: 10.1038/s41598-017-10351-9 (PMC5575150; doi:10.1038/s41598-017-10351-9)
Supplement: Supplementary file 1 — Supplemental Information [file 41598_2017_10351_MOESM1_ESM.pdf]

Supplementary Information

Identification of the protective mechanisms of Lactoferrin in the irradiated salivary gland

Manabu Sakai[1,2], Takumi Matsushita[1,3], Ryoko Hoshino[1], Hitomi Ono[1], Kazuki Ikai[1] and Takayoshi Sakai[1]*

[1] Department of Oral-facial Disorders, Osaka University Graduate School of Dentistry, 1-8 Yamadaoka, Suita, Osaka 565-0871, Japan
[2] Department of Clinical Laboratory, Osaka University Dental Hospital, 1-8, Yamadaoka, Suita, Osaka 565-0871, Japan
[3] First Department of Oral and Maxillofacial Surgery, Osaka Dental University, 8-1 Hanazono-cho, Kuzuha, Hirakata, Osaka 573-1121, Japan

* Correspondence should be addressed to Takayoshi Sakai; sakai@dent.osaka-u.ac.jp

Supplementary Figure and Figure legends

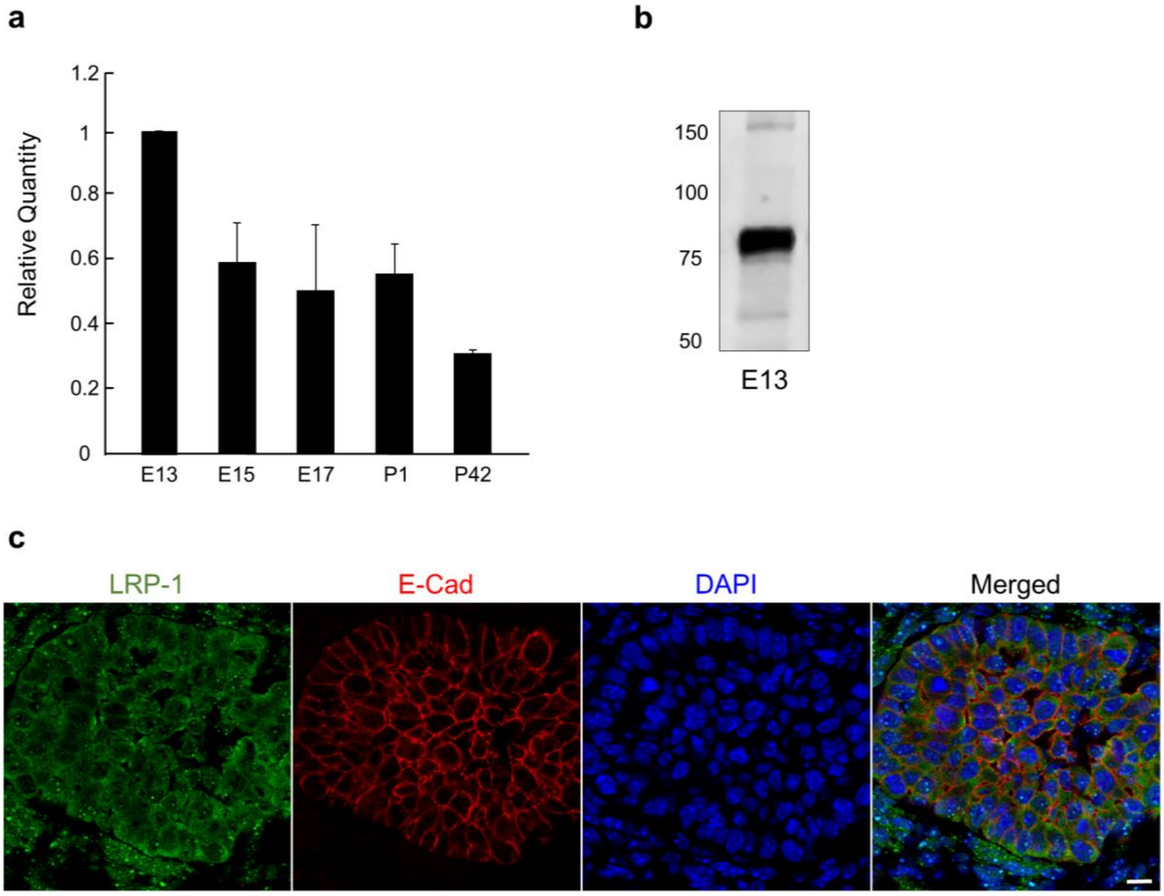

Figure S1. LRP-1 expression and localization of LRP-1 and E-Cadherin in SMGs. The expression LRP-1 was determined by qPCR analysis (a) and Western immunoblotting (b). LRP-1 mRNA expression levels in E13, E15, E17, P1 and P42 were normalized to that in E13 (a) and LRP-1 protein expression level in E13 (b). Bars represent the mean ± SEM. LRP-1 expression in E13 SMG was shown by confocal immunofluorescence microscopy imaging of staining for LRP-1, E-Cadherin, and DAPI (c). The three fluorescence images of the same confocal section are merged. Scale bar: 10 µm

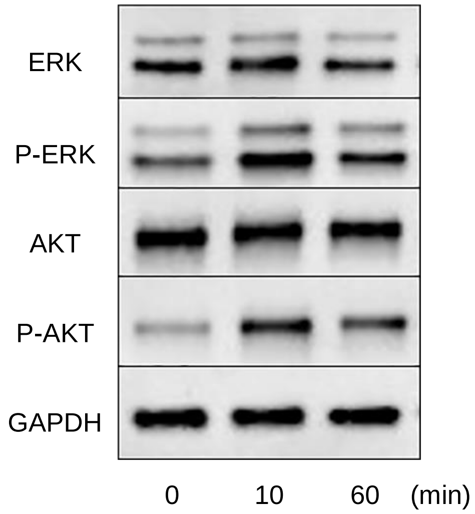

Figure S2. LF increased ERK1/2 and AKT phosphorylation in *ex vivo* organ culture.
ERK1/2 and AKT phosphorylation in E12.5 SMGs cultured for 72 h at 0, 10, and 60 min of culture with 0.1 mg/ml LF was analyzed by Western immunoblotting.

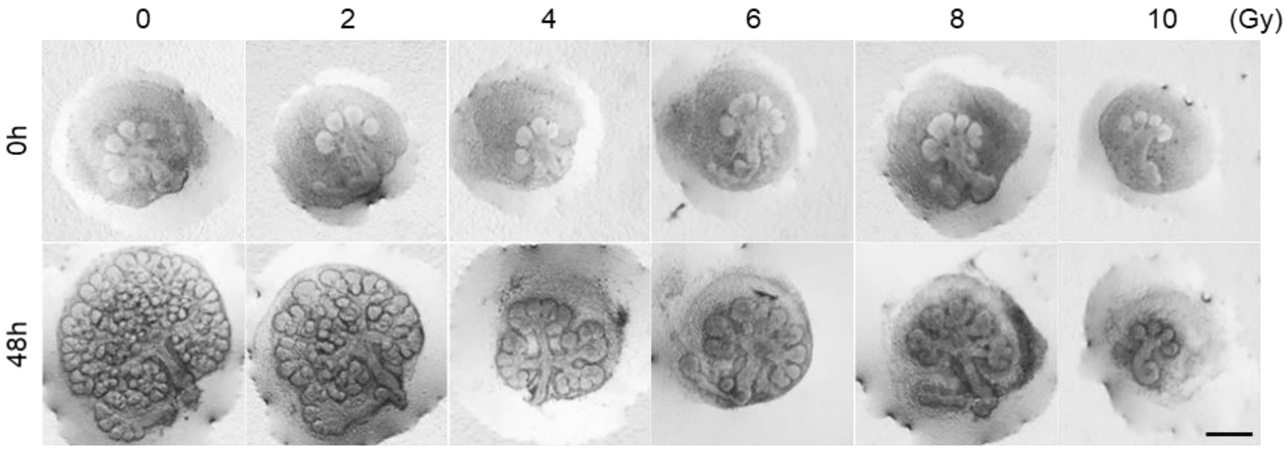

Figure S3. Irradiation altered the morphology of SMGs in a dose-dependent manner in *ex vivo* organ culture.
Phase-contrast images show E12.5 SMGs cultured for 24 h at 0 and 48 h after irradiation (2-10 Gy). Scale Bar: 500 µm.

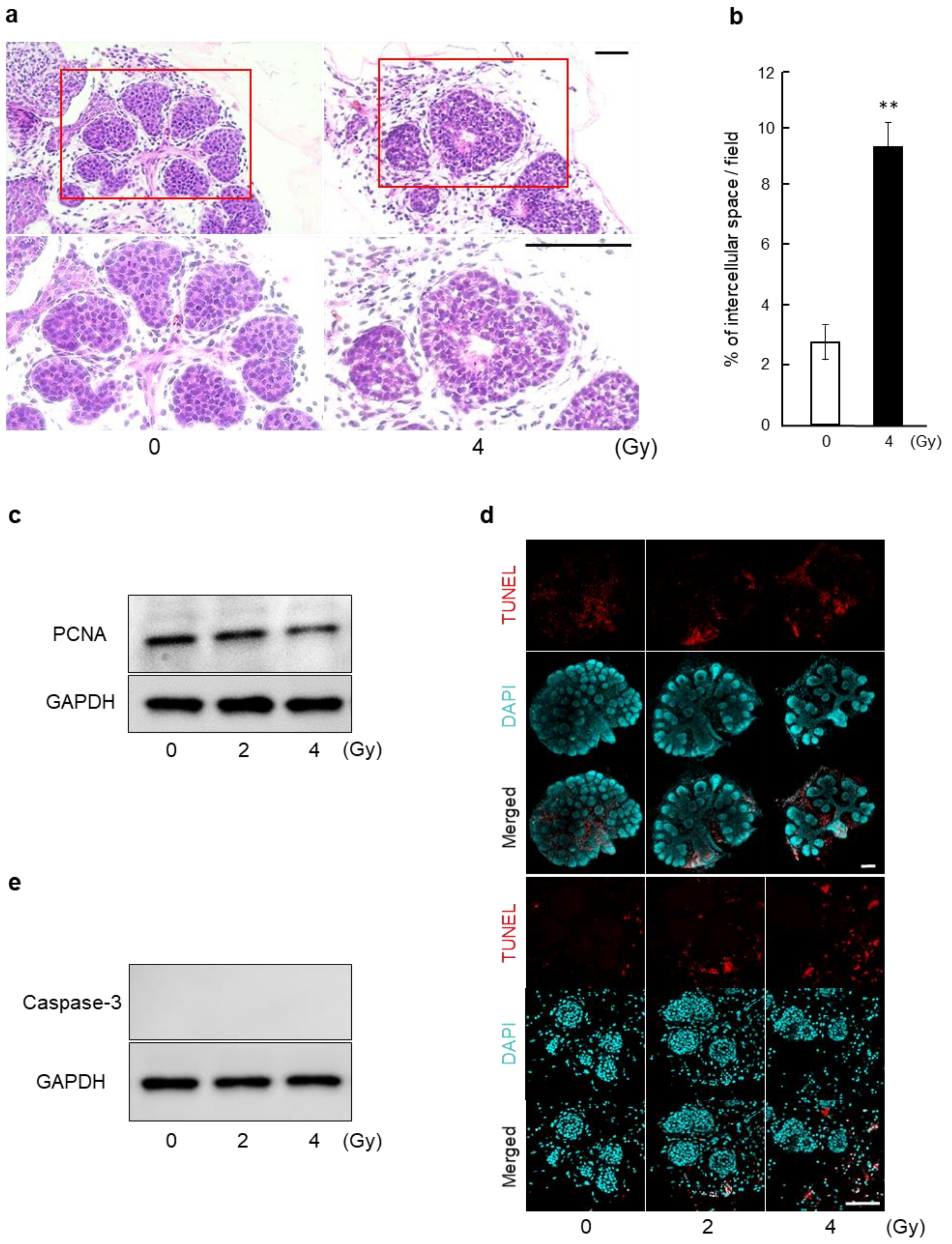
a
0
4
(Gy)
b
% of intercellular space / field
0
2
4
6
8
10
12
**
0
4
(Gy)
c
PCNA
GAPDH
0
2
4
(Gy)
d
TUNEL
DAPI
Merged
TUNEL
DAPI
Merged
0
2
4
(Gy)
e
Caspase-3
GAPDH
0
2
4
(Gy)

Figure S4. Effect of Radiation on both cell proliferation and apoptosis during SMG branching morphogenesis in *ex vivo* organ culture.

HE staining images show E12.5 SMGs cultured for 24 h at 48 h after irradiation (4 Gy) (a). Scale bar: 100 µm. The bottom pictures in A are higher magnifications of the top pictures, respectively. Quantification of the percent area per field (100 μm^3/field) occupied by intercellular space is graphed (b). PCNA (Proliferating cell nuclear antigen: cell proliferating marker) expression in E12.5 SMGs cultured for 24 h at 48 h after irradiation (2-4 Gy) was analyzed by Western immunoblotting (c). Apoptotic cells in E12.5 SMGs cultured for 24 h at 48 h after irradiation (2-4 Gy) were detected by TUNEL. DAPI was used to stain cell nuclei (d). The bottom nine pictures are higher magnifications of the top nine pictures, respectively. Scale bar: 150 µm. Active-Caspase-3 (cell apoptosis marker) expression in E12.5 SMGs cultured for 24 h at 48 h after irradiation (2-4 Gy) was analyzed by Western immunoblotting (e).

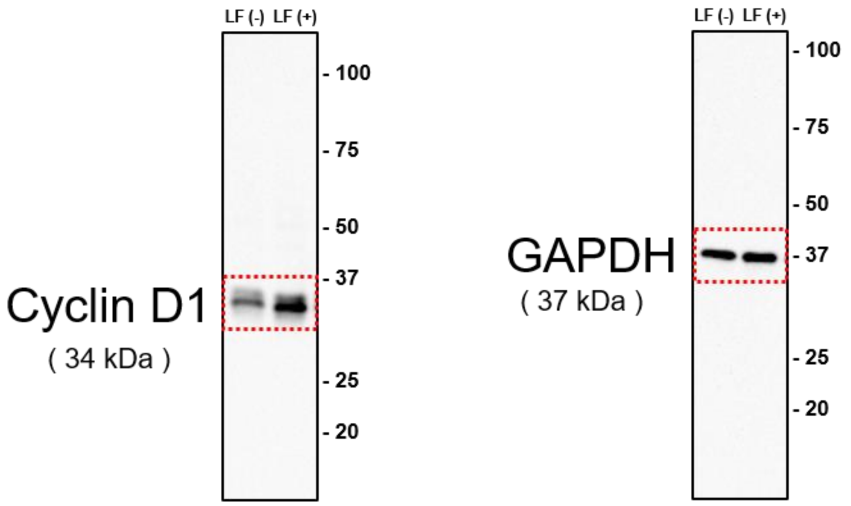

Figure S5. Full-length pictures of Western immunoblotting.

Full-length blots for Fig. 1q. Dashed red boxes indicate areas that were cropped. Numbers on right of image indicate positions of molecular weight markers (kDa).

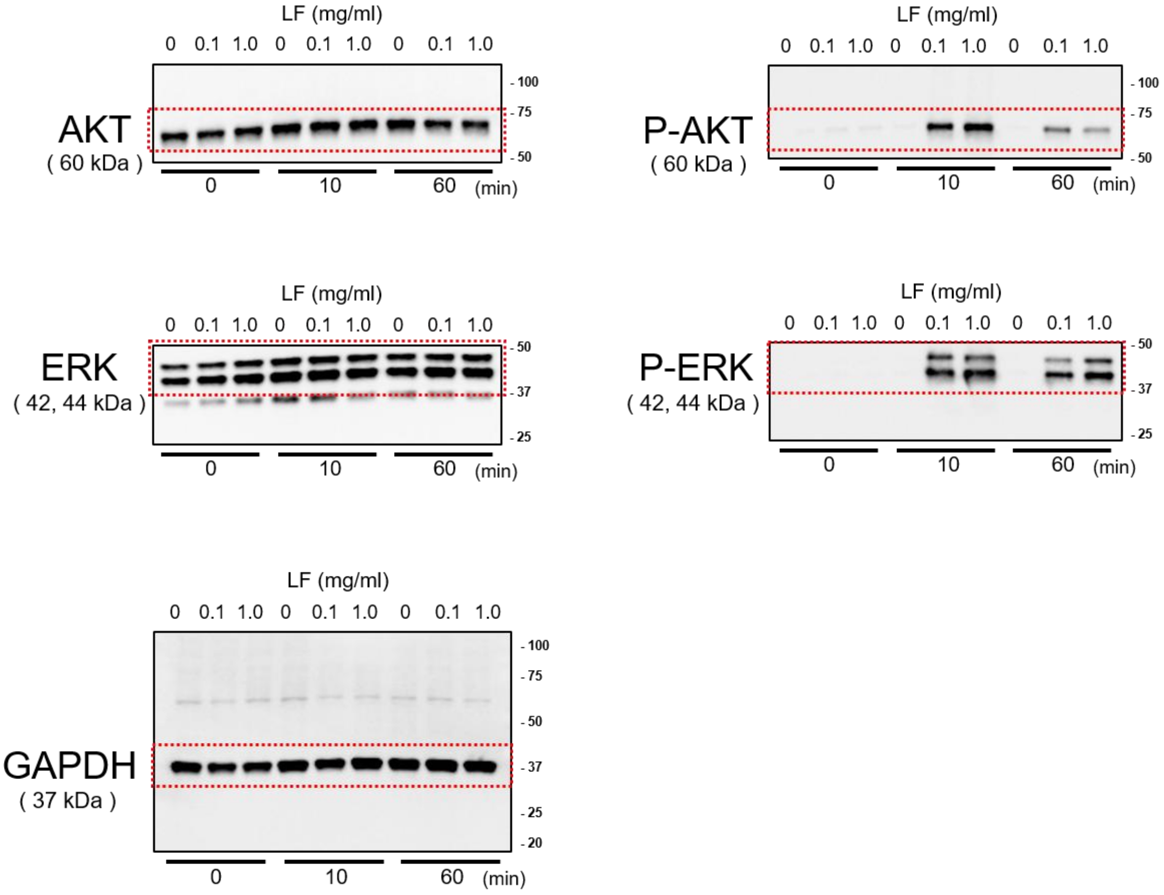

Figure S6. Full-length pictures of Western immunoblotting.

Full-length blots for Fig. 2. Dashed red boxes indicate areas that were cropped. Numbers on right of image indicate positions of molecular weight markers (kDa).

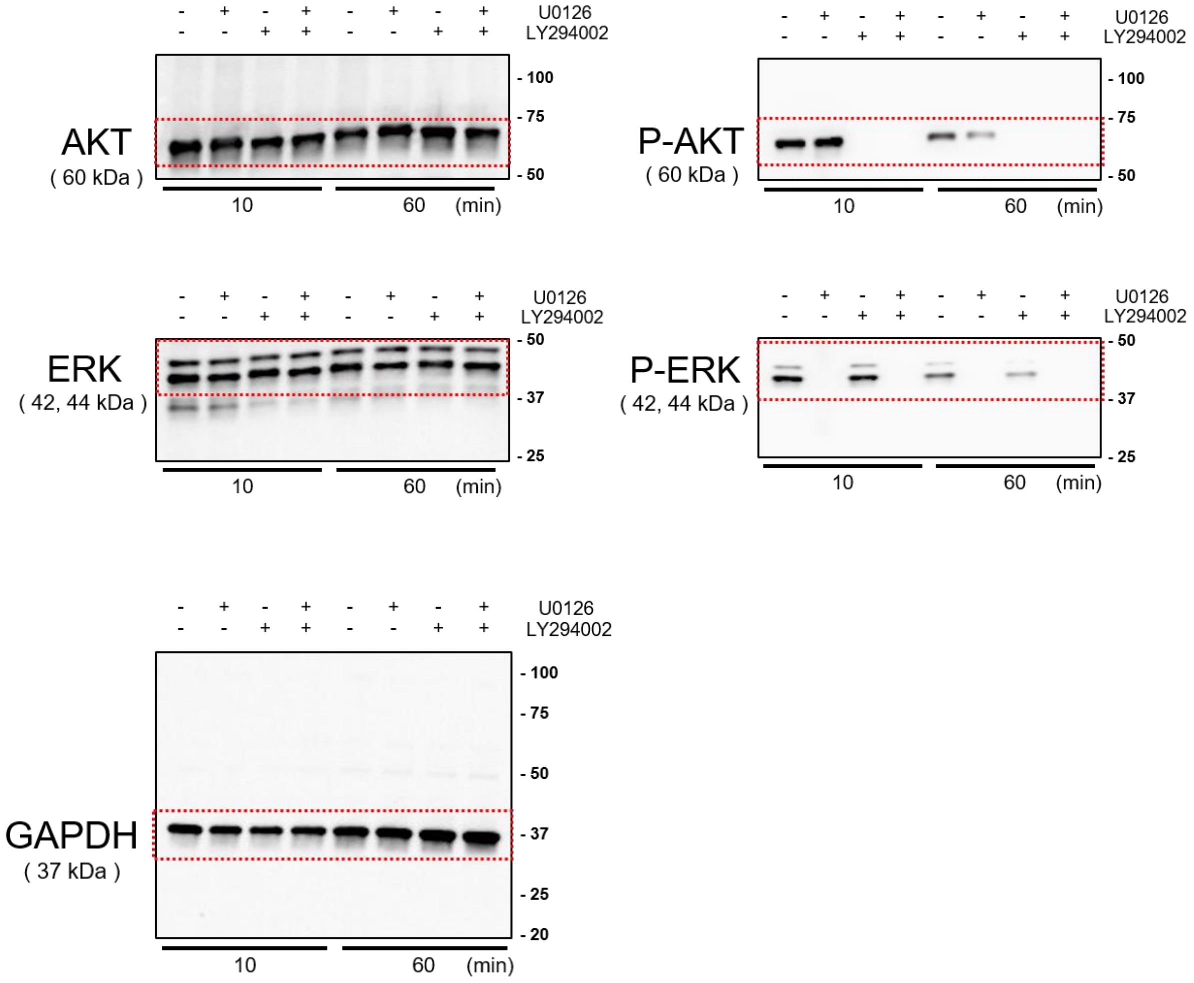

Figure S7. Full-length pictures of Western immunoblotting.

Full-length blots for Fig. 3i. Dashed red boxes indicate areas that were cropped. Numbers on right of image indicate positions of molecular weight markers (kDa).

Supplementary Methods

Cell death assay

Apoptosis was detected by TUNEL using an In Situ Cell Death Detection Kit TMR-red (Roche Applied Science, Indianapolis, IN, USA). The SMGs were fixed with 2% paraformaldehyde for 60 min, permeabilized with 0.1% Triton X-100 in 0.1% sodium citrate PBS on ice for 2 min, and incubated with a TUNEL reaction mixture at 37°C for 1 h. The SMGs were also stained with DAPI (1:500, Thermo Scientific, Waltham, MA, USA) to stain cell nuclei. Immunofluorescence was examined using SP8 confocal microscopy (Leica, Wetzlar, Germany).
